# Attentional bias and math avoidance: insights from a developmental sample

**DOI:** 10.1007/s00426-025-02089-1

**Published:** 2025-03-06

**Authors:** Alessandro Cuder, Eleonora Doz, Orly Rubinsten, Maria Chiara Passolunghi, Sandra Pellizzoni

**Affiliations:** 1 Department of Life Sciences, University of Trieste, Via Weiss n. 21, Trieste, Italy; 2 Department of Learning Disabilities, University of Haifa, Haifa, Israel

## Abstract

Stimuli perceived as threatening subtly influence how individuals orient their attention, a phenomenon labelled as attentional bias. According to literature, individuals with negatives attitudes toward math would exhibit attentional bias when presented with math-related stimuli. However, attentional bias and its relationships with math anxiety, math self-efficacy, and math skills are understudied, particularly when considering developmental samples. For this reason, the aim of the present study was to assess attentional bias toward math stimuli (i.e., math vs. neutral words) and to evaluate its relationship with math anxiety, math self-efficacy and math skills in fifth and sixth grade students (M_months_ = 135.84; SD_months_ = 7.53) tested in January 2023. Findings indicated that children who were more anxious and had lower levels of math self-efficacy and math skills appeared to avoid math stimuli in an attentional bias task. Furthermore, dominance analysis showed that math self-efficacy made the largest average contribution in attentional bias scores, suggesting that motivational constructs would play a central role in the observed attentional bias avoidance patterns. Results could potentially generalize developmental age samples, providing new insight into how avoidance behaviors, even for stimuli that are not purely numerical, would influence children’s attentional processes rapidly and automatically, posing a risk factor for maintaining negative attitudes toward math.

## Introduction

In an increasingly technological and data-driven world, mathematical knowledge is an interpretive tool that guides individuals and societies to understand increasingly complex realities and keep up with the technological and scientific developments of today’s times (Gerardi et al., 2013; Gross et al., 2009; Rivera-Batiz, 1992). A decades-long strand of research in math learning has shown how affective-motivational factors and math skills are associated to individuals’ attitudes towards math and future academic and career choices (Ahmed, 2018; Choe et al., 2019; Cuder et al., 2024; Daker et al., 2021; Huang et al., 2019; Krinzinger et al., 2009). However, only recently, scientific debate has pointed out how individuals’ negative attitudes toward math would also result in a subtle alteration of attentional processes fundamental to the processing of math stimuli (Pizzie & Kraemer, 2017; Rubinsten et al., 2015), phenomenon that goes by the name of attentional bias (MacLeod et al., 1986; Mogg et al., 2004). The present study represents one of the first attempts to investigate how affective-motivational factors and math skills would influence attentional biases toward math stimuli in children attending primary and middle school.

### Affective-motivational factors and math skills

As early as in primary school, it is well known that affective-motivational factors and math skills interact with each other influencing math learning. Among affective factors, math anxiety (MA) is defined as a feeling of tension that interferes with the manipulation of numbers and the solving of arithmetical problems (Richardson & Suinn, 1972). This form of anxiety is usually evoked in situations involving math, affecting learning as early as primary school (Pellizzoni et al., 2022; Ramirez et al., 2016) and leading to lower math skills (Barroso et al., 2021; Caviola et al., 2022; Foley et al., 2017; Namkung et al., 2019) and lower math attitudes (Ahmed et al., 2012; Cuder et al., 2024; Li et al., 2021), such as math self-efficacy (Justicia-Galiano et al., 2017; Li et al., 2021). The involvement of MA is pervasive, influencing, for instance, the judgment of the threateningness of math words (Layzer Yavin et al., 2022) and future academic and job choices (Ahmed, 2018; Choe et al., 2019; Cuder et al., 2024; Huang et al., 2019; Quintero et al., 2022).

Math self-efficacy, on the other hand, indicates a set of beliefs about one’s perceived competence with respect to solving math tasks and succeeding in math (Bandura, 1996; Di Giunta et al., 2013; Pastorelli et al., 2001). Individuals with higher math self-efficacy would show a greater sense of competence, interest in math, perseverance in the face of difficulties and engagement in math (Ainley & Ainley, 2011; Grigg et al., 2018; Levine & Pantoja, 2021; Lopez & Lent, 1992; Martin & Rimm-Kaufman, 2015). In turn, affective-motivational aspects seem to have a bidirectional relationship with math skills, consequently modulating attitudes towards math (e.g., Carey et al., 2016; Levine & Pantoja, 2021; Živković et al., 2023). In this context, having high MA, low math self-efficacy and poor math skills represents a risk factor for the development of negative attitudes towards math stimuli as well as influencing basic cognitive processes responsible for processing them (Hunt et al., 2015; Levine & Pantoja, 2021; Ramirez et al., 2013; Rubinsten et al., 2015; Suárez-Pellicioni et al., 2014).

### The attentional bias

Several studies have shown how attentional processes can be altered when the individual is exposed to threatening stimuli (MacLeod et al., 1986; Mogg et al., 2004). According to the literature, attentional bias is thought to manifest through both vigilance and avoidance patterns (Abado et al., 2020; Mogg et al., 2004). Vigilance involves attentional engagement with a stimulus perceived as threatening, whereas avoidance is characterized by attentional disengagement from the threatening stimulus. These patterns are particularly evident in experimental paradigms such as the Dot-Probe task, where threatening and neutral stimuli are presented in different spatial locations. A probe (i.e., the target stimulus) is then presented in the same or a different location relative to the previously presented threatening or neutral stimuli. Reaction times (RTs) to the probe are compared between neutral and threatening conditions, allowing researchers to infer whether a vigilance or avoidance pattern is present. Specifically, in the case of vigilance, individuals are expected to respond more quickly to a probe that appears in the same location as the previously presented threatening prime. In contrast, avoidance is characterized by slower responses to probes appearing in that same location, as individuals must reorient their attention to the target after initially diverting it away from the threatening prime (Pizzie & Kraemer, 2017). From a theoretical point of view, attentional bias would also occur with a time course (Abado et al., 2020; Mogg et al., 2004). Specifically, individuals would exhibit an immediate pattern of vigilance when exposed to threatening stimuli as opposed to neutral stimuli. Following this rapid engagement, individuals would shift their attention away from the threatening stimulus, showing an avoidance pattern. In this context, it is argued that the evaluation of the threatening stimulus could be guided at first by processes which would allow rapid identification of the threat. Subsequently, the threatening stimulus would be processed by mechanisms that would lead the individual to manifest avoidance patterns (Cisler & Koster, 2010; Bar-Haim et al., 2007). In this line, it has been proposed that vigilance and avoidance patterns would be functional in quickly detecting threat and then pursuing emotional regulation strategies (Cisler & Koster, 2010).

The etiology and maintenance of anxiety disorders have been shown to be significantly influenced by attentional bias (for review, see Abado et al., 2020; Okon-Singer, 2018; Putwain et al., 2020). Although it also exists in healthy populations, attentional bias is more pronounced in clinical and subclinical populations (for reviews, see Abado et al., 2020; Aue & Okon-Singer, 2015). For instance, attentional bias has been observed in association with various anxiety-related disorders (e.g., Rosen et al., 2019; Salum et al., 2013; Waters et al., 2014) but also in relation to motivational constructs (Karademas et al., 2007). For example, considering developmental stages, a study by Salum and colleagues (2013) showed that attentional bias toward threatening stimuli can vary according to the type of disorder (e.g., stress or fear disorder) and the severity of symptoms. Similarly, in a study by Waters and colleagues (2014), attentional bias was observed in children with anxiety disorders, revealing a vigilance pattern toward threats in children with generalized anxiety disorder and an avoidance bias in those with fear-related disorders. Finally, a meta-analysis by Dudeney and colleagues (2015) examined attentional bias towards threatening stimuli in children with anxiety, concluding that anxious children show a greater attentional bias compared to non-anxious children, and that this difference increases with age. When it comes to math learning, individuals with high MA would exhibit attentional bias toward math stimuli perceived as threatening (Cohen & Rubinsten, 2017; Eidlin Levy et al., 2021; Rubinsten & Tannock, 2010) showing vigilance (e.g., Rubinsten et al., 2015) or avoidance (Pizzie & Kraemer, 2017) patterns. Furthermore, attentional bias toward math stimuli has been little investigated in relation to relevant aspects that might influence it, such as motivational factors.

Evidence suggests that MA and math self-efficacy are two constructs that negatively and reciprocally influence each other (Ahmed et al., 2012; Du et al., 2021), highlighting the importance of simultaneously investigating both factors in relation to attentional bias. For instance, in a study by Karademas and colleagues (2007), the authors found that constructs such as dispositional self-efficacy and optimism influenced attentional bias toward positive and threatening stimuli. Studies suggest that in the later stages of attentional bias, top-down regulation processes may come into play, leading individuals to avoid stimuli perceived as threatening (for a review, see Cisler & Koster, 2010). Indeed, evidence seems to show that motivational factors may play the role of proactive control processes of attention, consequently influencing how the individual directs attention to stimuli with emotional valence (e.g., Walsh et al., 2018). In other words, motivational factors could be involved in the modulation of attentional processes, especially in the later stages, when emotional stimuli are processed by regulatory and more controlled attentional mechanisms. (Cisler & Koster, 2010). In the present study, we chose to assess math self-efficacy because, in addition to its common association with avoidance behaviors in mathematics (Cuder et al., 2024; Lau et al., 2024), it provides a more specific measure of the child’s perceived mastery in solving math tasks. This approach offers a more direct and focused assessment of the child’s motivation in math compared to broader motivational constructs (e.g., math self-concept), which would require the child to reflect on their past experiences in math, their skill level relative to peers, and performance in other school subjects (Möller & Marsh, 2013; Müller-Kalthoff et al., 2017).

Considering developmental samples, several investigations suggest that attentional biases may occur for various phobic and distress disorders in primary and middle school students (see Salum et al., 2013). In a study conducted by Haft and colleagues (2019), the authors found that children with specific learning disorders showed a pattern of avoidance to words semantically related to reading. However, to date, it remains unclear whether attentional bias toward math stimuli can also occur in school-age children. Indeed, starting in primary school, children may begin to develop negative attitudes toward math from MA, low motivation, or poor math skills (e.g., Caviola et al., 2022; Živković et al., 2023). These negative attitudes, consequently, would affect the way math stimuli are processed at the attentional level.

### The present study

In light of the theoretical framework, it is relevant to investigate whether children’s affective-motivational factors and math skills may influence attentional bias toward math stimuli. As a result, the aims of the present study were twofold: (1) evaluate how MA, math self-efficacy and math skills influence attentional bias, exploring vigilance and avoidance patterns toward math words (vs. neutral) in a sample of primary and middle school students; and (2) explore the unique contribution of MA, math self-efficacy and math skills on attentional bias toward math stimuli.

Regarding the assessment of attentional bias, we made no specific assumptions about its directionality (i.e., patterns of vigilance-avoidance to threat stimuli) given that the literature is unclear on whether individuals would show vigilance (e.g., Rubinsten et al., 2015) or avoidance (Pizzie & Kraemer, 2017) patterns toward math stimuli, especially when considering primary and middle school students. We hypothesized that children’s attentional bias toward math words would be related to MA and math skills. Indeed, previous research on adult populations showed that MA and math skills would influence attentional bias toward math stimuli (Cohen & Rubinsten, 2017; Eidlin Levy & Rubinsten, 2021; Pizzie & Kraemer, 2017; Rubinsten et al., 2015). We also expected to find an effect of math self-efficacy on attentional bias toward math stimuli. Indeed, some evidence found that motivational factors are associated to attentional bias (e.g., Karademas et al., 2007), influencing how individuals’ direct attention towards positive and negative stimuli (Cisler & Koster, 2010; Padmala et al., 2017; Pourtois et al., 2013; Vogt et al., 2020; Walsh et al., 2018).

The attentional bias is a complex phenomenon that is influenced by affective-motivational factors and basic cognitive skills (Eidlin Levy & Rubinsten, 2021; Karademas et al., 2007; Pizzie & Kraemer, 2017; Rubinsten et al., 2015). In this context, we planned to conduct a dominance analysis, which would identify predictors’ relative importance in a statistical model (Budescu, 1993) and could supplement multiple regression analysis. The relative importance of predictors in a multiple regression analysis is typically determined by the magnitude of the effects expressed through the regression coefficients. However, these can fluctuate with the inclusion of other predictors in the model. To address this, dominance analysis estimates the relative importance of a predictor by assessing the average additional variance it contributes to explaining the dependent variable, accounting for all possible predictors’ combinations (see the “Results” section for a detailed explanation of the statistical procedure). By assessing a variable’s average added contribution across various submodels, dominance analysis, in conjunction with regression modeling (Azen & Budescu, 2003), allows for a more nuanced identification of the predictors most strongly associated with attentional bias considering MA, math self-efficacy and math skills.

The present study aimed at expanding previous literature by introducing some theoretical and methodological novelties. First, we considered within the same study the contribution of both affective-motivational aspects (i.e., MA and math self-efficacy) and math skills on attentional bias to math stimuli. To the best of our knowledge, this represents the first attempt to investigate the relationship between math self-efficacy and attentional bias toward math stimuli. Investigating motivational aspects such as math self-efficacy is crucial because theoretically, they may play a role in explaining the vigilance and avoidance processes that concur to influence attentional bias as suggested by previous studies (e.g., Karademas et al., 2007). Secondly, the current study advances existing literature by investigating the attentional bias toward math stimuli in primary and middle school students. In fact, previous studies mainly focused on older students and adults (Cohen & Rubinsten, 2017; Pizzie et al., 2017; Rubinsten et al., 2015), neglecting however younger students (i.e., primary and middle school students) who are still in the process of learning math. The decision to focus specifically on this age group lies in the evidence showing the transition from primary to middle school as a period in which mathematical curricular demands increase, which consequently affects affective-motivational aspects as well (e.g., Namkung et al., 2019). Additionally, it should be noted that in the present study, we employed a Dot-Probe task (see Rubinsten et al., 2015) to assess attentional bias toward math words instead of numbers or math operations, as numerous studies have demonstrated that negative (Citron et al., 2014; Sutton & Lutz, 2019) and math (Cohen et al., 2021; Layzer Yavin et al., 2022) words are emotionally evocative. The Dot-Probe task involves presenting participants with prime stimuli that can be either emotionally salient or neutral, followed by a probe that appears in the same or different location as the prime. Response times to the probe are then measured to assess participants’ attentional bias toward the prime stimuli (see the “Materials” section for a detailed description of the task). Previous studies have exposed participants to both emotionally salient math-related words and math operations (e.g., Daches Cohen & Rubinsten, 2017; Rubinsten et al., 2015) to measure attentional bias. Since our study focuses on students in developmental stages, we simplified the task by exposing participants only to math-related words, as done in a previous study by Haft and colleagues (2019). Finally, since some evidence indicated that general forms of anxiety may influence attentional bias (Dudeney et al., 2015; Salum et al., 2013; Waters et al., 2014), we controlled for general anxiety to avoid possible confound effects in our analyses.

## Method

### Participants

Prior to conducting the study, we performed a priori power analysis using G*Power (Faul et al., 2007). Specifically, in light of the aims of the study, the dominance analysis resulted in the specification of a set of sub-models, with the most complex being a regression model featuring five predictors (i.e., age, general anxiety, MA, math self-efficacy, and math skills) for the attentional bias scores. Consequently, we performed a power analysis based on a regression model with five predictors, adopting an alpha level of 0.05, a statistical power of 0.80, and a moderate effect size from the predictors (*f*^*2*^ = 0.15). The analysis showed that the appropriate sample size was 55 participants.

A sample of 66 students from the fifth grade of primary school and first grade of middle school were recruited into the study. Subsequently, three students were excluded from the analyses for exhibiting poor accuracy in the Dot-Probe task (*n* = 1 for accuracy below 80% in probe identification; *n* = 2 for accuracy below 80% in rhymes), and two for having outlier math skills. Thus, the final sample consisted of 61 children (M_months_ = 135.84; SD_months_ = 7.53; age range = 117–151 months; F = 27; M = 34) attending the 5th (*n* = 25) and 6th grade (*n* = 36). A student did not participate in the math skills assessment and was therefore excluded from the analyses through listwise deletion when this specific variable was considered. The participants were all Caucasian, of average socio-economic status according to schools’ records. Five students (*n* = 5, 8.20% of the total sample) had an immigrant background from other EU countries, consistent with national data regarding the foreign composition of the student population. Following the approval of the principal and teachers to participate in the research project, parents gave written consent for their children to participate in the study. Students were informed that their participation was voluntary and that they could withdraw at any time. Before the assessments, children were verbally asked to consent to participating in the activities. The study was approved by the ethics committee of the University of Trieste. The study was conducted in line with the Declaration of Helsinki and the ethical guidelines of the Italian Association of Psychology.

### Procedure

The tests were administered in January 2023, conducting two sessions close in times. In the first session conducted collectively in the classroom, students were administered the questionnaires and the math tasks. In the second session, conducted individually with each student in a quiet space within the school, the Dot-Probe task was administered in computerized form.

### Materials

#### Dot-probe task

**Fig. 1:**
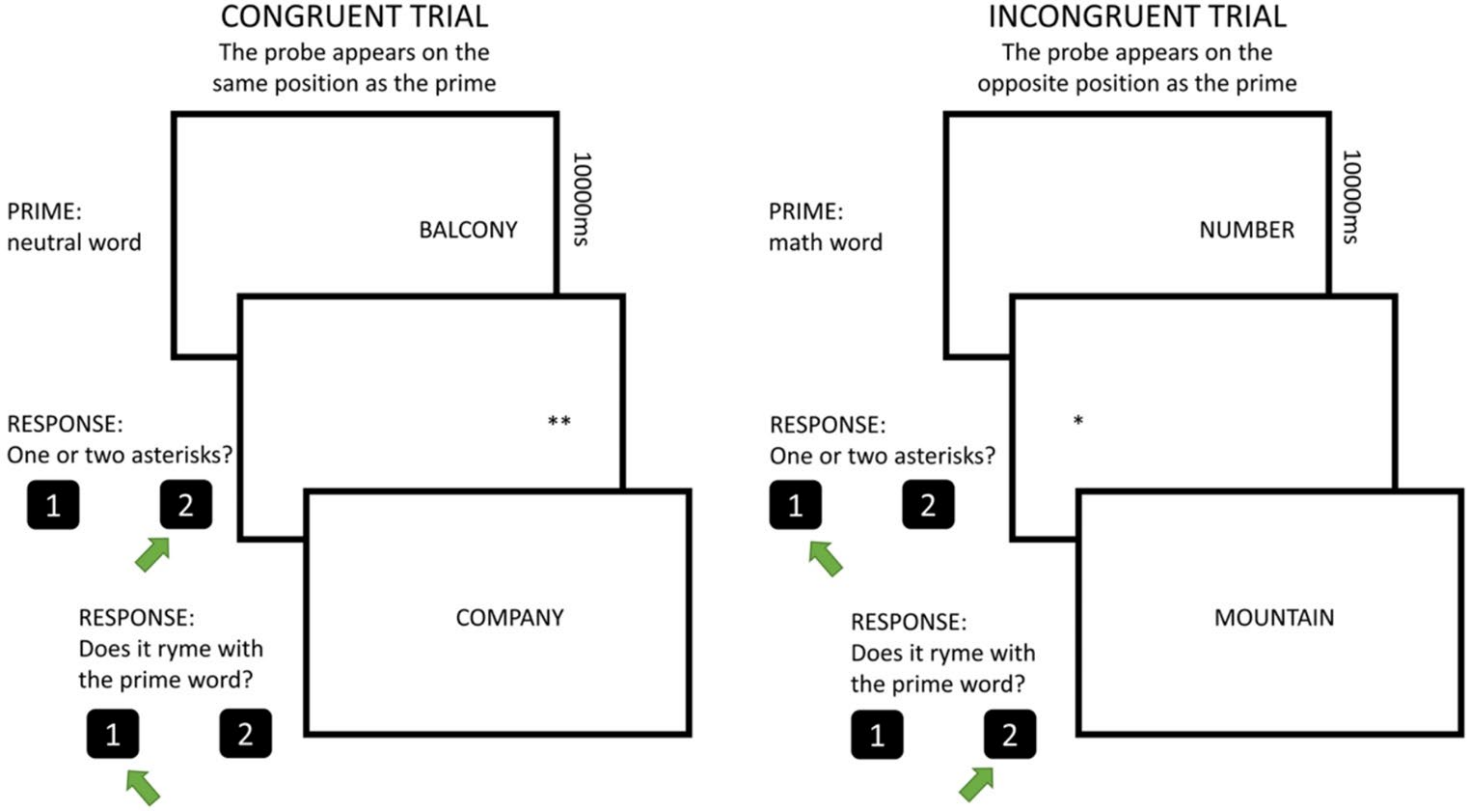
Graphical representation of the Dot-Probe task. Both the prime words and the words used in the rhyme test were presented in Italian

An adaptation of the Dot-Probe task (MacLeod et al., 1986; Rubinsten et al., 2015) was used to measure attentional bias toward math words (Fig. 1).

The instrument was developed using the Experiment Builder Psychopy (Peirce et al., 2019) and adapted from a paradigm originally designed for adult participants (Rubinsten et al., 2015). The task consisted of four blocks: one practice block (10 trials) and three evaluation blocks (16 trials each). The word stimuli consisted of 16 math words and 16 neutral words. Following the method used by Rubinsten et al. (2015), the word stimuli used in the study were selected based on a pilot study conducted on an independent sample of primary and middle school students (*n* = 105). Specifically, we first selected a list of 16 math words and a list of 16 neutral words using a frequency lexicon of the Italian language (Bertinetto et al., 2015). Next, we checked that the selected words did not differ in number of syllables and letters. Finally, we constructed a self-report questionnaire, in which we asked each child to rate the degree of perceived familiarity with respect to the words (i.e., math and neutral words). The results showed that math and neutral words did not differ in degree of perceived familiarity.

In this task, each trial began with a fixation point presented for 740ms followed by a blank screen presented for 100ms. Next, a word stimulus semantically associated with math (e.g., “number”) or neutral (e.g., “balcony”) was presented in the central left or central right part of the screen for 1000ms. This was followed by an inter-stimulus interval that could randomly range from 100ms to 150ms. The random duration of the inter-stimulus interval prevented participants from predicting the temporal appearance of the probe (Posner & Boies, 1971). Next, a probe was presented (either one “*” or two asterisks “**”) that appeared in the same portion of the screen as the word stimulus (congruent condition) or opposite (incongruent condition). Participants had to respond on a QWERTY keyboard with “1” if there was only one asterisk and “2” if there were two. The probe remained on the screen for 3000ms or until the participant provided the answer. To ensure that children had processed the prime correctly, a second word would appear after the probe, which may or may not rhyme with the previously presented prime word. In case it rhymed, the participant had to respond with “1” otherwise with “2”. Both the prime words and the words used in the rhyme test were presented in Italian. After the answer or 4000ms a blank screen would appear for 1500ms, and the next trial would begin. There was a one-minute break between the three evaluation blocks (Fig. 1).

Before calculating the Dot-Probe task scores, trials in which the participant responded incorrectly to the probes or to the rhymes were removed from the analyses. This procedure was adopted to avoid conducting analyses on careless responses from participants and to ensure that they had properly processed the prime word. The split-half reliabilities computed on the RTs to the probes, were adequate for math (*r* =.92) and neutral words (*r* =.81) in the congruent condition, and for math (*r* =.90) and neutral words (*r* =.73) in the incongruent condition.

In this task, we assess how participants direct their attention toward threatening (math) prime words by comparing their RTs to a neutral prime presentation (see Pizzie & Kraemer, 2017; Haft et al., 2019). We analyzed two conditions: a congruent one, where the probe appears in the same location as the prime, and an incongruent one, where the probe appears on the opposite side of the screen. In the congruent condition, if participants respond faster to a probe that follows a threatening math prime (compared to a neutral prime), we interpret this as a vigilance bias: the threatening prime biases participants’ attention in that location, facilitating faster detection of the following probe. However, if participants respond more slowly to the probe in the same spot as the threatening math prime, we interpret this as an avoidance pattern. In other words, once they are exposed to the threatening prime, they shift their attention away from its location, causing a delay in responding to the probe. In the incongruent condition, if participants respond faster to a probe on the opposite side of the screen from a threatening math prime (compared to a neutral prime), we interpret this as an avoidance pattern, suggesting that the threatening prime prompted them to disengage attention from its original location and shift it to the other side of the screen, where the probe would appear. Conversely, if participants respond more slowly to the probe in the incongruent condition, we interpret this as a vigilance pattern, implying that the threatening prime biased participants’ attention, thus requiring more time to reorient toward the probe’s location on the other side of the screen. In this context, Dot-Probe task scores were calculated by subtracting the RTs for the probes that were preceded by a math word from the RTs for the probes preceded by a neutral stimulus word for the congruent (congruent Dot-Probe task) and incongruent (incongruent Dot-Probe task) condition, respectively. In the congruent condition, higher Dot-Probe task scores would indicate a vigilance response to math words compared to neutral words, whereas lower scores suggest an avoidance pattern toward math-related words. On the other hand, in the incongruent condition, higher scores reflect an avoidance pattern toward math words, while lower scores would suggest a vigilance response to math-related words compared to neutral ones.

#### Math anxiety

The Abbreviated Math Anxiety Scale (AMAS, Hopko et al., 2003, Italian version adapted by Caviola et al., 2017) was used to measure MA. The questionnaire consisted of nine self-report items, in which students were asked to rate the degree of fear they felt in certain situations involving math (e.g., “Using the diagrams and tabs reported at the back of the math book”, “Thinking about the written math test you will have to do tomorrow”, or “Taking a written math test”). The response was given on a Likert scale from 1 (i.e., “very little fear”) to 5 (i.e., “very much fear”). The total score was equal to the sum of the points given on the Likert scale (score range: 9–45). The instrument shows good reliability in our study (Cronbach’s alpha = 0.76) and in validation studies (Cronbach’s alpha = 0.77; Caviola et al., 2017).

#### Math Self-Efficacy

To measure math self-efficacy, we administered a 5-item self-report questionnaire (adapted from Di Giunta et al., 2013) asking students to rate their ability to deal with various situations involving math. Items included “How good you are at learning math”; “ How good are you at mental calculation”; or “How good you are at solving column operations”. Participants responded using a 5-point Likert scale, where 1 corresponds to low perceived effectiveness (i.e., “Not good at all”) and 5 to high perceived effectiveness (i.e., “Very good”). The total score was the sum of all scores (score range: 5–25). Observed reliability is good in the present study (*Cronbach’s alpha* = 0.73) and in the literature (*Cronbach’s alpha* = 0.77; Cuder et al., 2024).

#### Math skills

Regarding the assessment of math skills, some tasks of the AC-MT battery (AC-MT-3 6–14, Cornoldi et al., 2020) were used. Three paper-and-pencil subtests were selected: approximate calculation, mathematical fluency, and inferences. In the approximate calculation test, students were asked to mentally solve 15 math operations in an approximate manner, indicating among three alternatives the one that was closest to the correct result. Participants were instructed not to perform the operation but to estimate its result and find the value that came closest to it. The test-retest reliability for the approximate calculation test was good according to the instrument manual (*r* =.73). In the fluency task, students had to solve 15 math operations presented in columns (i.e., addition, subtraction and multiplication) as fast as possible. The test-retest reliability for the fluency task is good according to the instrument manual (*r* =.89). In the inference task, the participant was asked to perform three different tasks each consisting of four items. The first, required the student to perform operations presented as symbol-number equivalences. For example, operations as “scissors + scissors = 4” were presented and participants were asked to identify the numerical value corresponding to the scissors symbol. In the second task, operations were presented in Arabic format. These were composed of the addends and the result but with the operator missing. The participants’ task was to correctly enter the sign of the operation, which corresponded to the presented result. Finally, the third task presented pairs of operations, one complete and one with no result. The two operations were very similar to each other, and the complete operation provided a useful clue for solving the incomplete one. In other words, participants were required to solve the incomplete operation, not by doing the calculation in their heads, but by helping themselves with the operation already performed. The inference test shows acceptable test-retest reliability according to the instrument manual (*r* =.69). All tasks were timed, and the duration was one and a half minutes for the approximate calculation task; one minute for the math fluency task; and two minutes for the inference task. One point was awarded for each correctly solved item, resulting in a final score that could range from 0 to 42 points.

#### General anxiety

General anxiety was measured with the Revised Children’s Manifest Anxiety Scale (RCMAS-2, Reynolds et al., 2012; Italian Edition), using the short version of the scale. The assessment instrument is self-report and involves 10 items with a binary response (i.e., “yes” or “no”). In responding to the items, students were asked whether the proposed situation describes their daily life experience. Some examples of the proposed items are “I often worry about something bad that might happen to me”, “Sometimes I wake up scared”, or “I have a feeling that someone will tell me I’m doing things wrong”. The total score corresponds to the sum of all affirmative answer (scoring range: 0–10). Reliability of the instrument is good in the present study (*Cronbach’s Alpha* = 0.74) and in the instrument manual (*Cronbach’s Alpha* = 0.82).

## Results

Data were analyzed using R, version 4.3.1 (R Core Team, 2023) and the package *dominanceanalysis*, version 2.1.0 (Bustos & Soares, 2024). The data supporting this study are openly available on the Open Science Framework (OSF) at https://www.org/osf.io/u7ehp/. Descriptive statistics and bivariate correlations are shown in Table 1.

**Table 1:** Descriptive statistics (mean; standard deviations; skewness and kurtosis) and bivariate correlations of the measures

Variable	M	SD	Skewness	Kurtosis	1	2	3	4	5
1. MA	20.48	5.23	0.42	-0.29	-	-	-	-	-
2. General anxiety	4.05	2.62	0.50	-0.84	0.36**	-	-	-	-
3. Math Self-efficacy	19.02	3.46	-0.51	-0.68	− 0.68**	− 0.25	-	-	-
4. Dot-Probe task (congruent)	-0.01	0.11	-0.41	1.08	− 0.27*	− 0.03	0.43**	-	-
5. Dot-Probe task (incongruent)	-0.01	0.12	0.03	1.13	0.18	0.19	− 0.15	− 0.19	-
6. Math skills	20.84	7.88	-0.02	-0.20	− 0.42**	− 0.15	0.47**	0.28*	− 0.11

Note. M = mean; SD = standard deviation

* *p* <.05; ** *p* <.01

Descriptive statistics for the dot-probe task reveal that participants showed good accuracy in identifying the probe (M = 0.89, SD = 0.07) and for the rhyme task (M = 0.85, SD = 0.11). Furthermore, they showed the following response times for the probe (M_RT(SECONDS)_ = 0.88, SD_RT(SECONDS)_ = 0.14) and the rhymes (M_RT(SECONDS)_ = 1.23, SD_RT(SECONDS)_ = 0.30).

Bivariate correlations show that Dot-Probe task scores in the congruent condition are negatively correlated with MA (*r* = −.27, *p* =.033), suggesting that greater MA is associated with increased avoidance of math-related word stimuli. Bivariate correlations also showed that Dot-Probe task scores in the congruent condition were positively correlated with math self-efficacy (*r* =.42, *p* =.001) and math skills (*r* =.28, *p* =.026), suggesting that higher math self-efficacy and math skills are associated with lower avoidance of math-related word stimuli. No correlation between Dot-Probe task scores in the incongruent condition and MA (*r* =.18, *p* =.177), math self-efficacy (*r* = −.14, *p* =.264) or math skills (*r* = −.11, *p* =.379) has been found, therefore we did not further consider this condition in the analyses. One possible explanation for the lack of correlation in the incongruent condition could stem from differences between our task and other Dot-Probe paradigms (e.g., Haft et al., 2019). In traditional Dot-Probe tasks, two prime stimuli are presented in distinct spatial locations, where the probe subsequently appears. This setup facilitates spatial detection of the probe, even in the incongruent condition. Our task, however, aimed to ensure that participants accurately processed the probe stimulus by presenting a single prime that was not spatially predictive of the probe’s appearance in the incongruent condition. In this context, the incongruent condition may have triggered alternative cognitive processes, such as the reorientation of attention towards the probe, resulting in less consistent patterns of attentional bias.

### Regression analysis

In order to evaluate the contribution of affective-motivational factors and math skills on attentional bias, three different multiple regression models were conducted (Table 2), placing Dot-Probe scores in the congruent condition as the dependent variable and age and general anxiety as covariates. In each regression model, MA (Model 1), math self-efficacy (Model 2) and math skills (Model 3) were placed as predictors. All predictors were included as continuous predictors and centered before conducting the regression analyses. We report the Bayes factor in favor of the alternative hypothesis (BF_10_) for each predictor, which we interpreted as supporting the alternative hypothesis over the null hypothesis.

**Table 2:** Regression analysis considering the dot-probe task scores in the congruent condition as dependent variable. In each model, either math anxiety (MA), math self-efficacy or math skills were placed as predictors. Age and general anxiety were considered as covariates

		β	SE	t	*p*	95% CI	BF_10_
Model 1	Intercept	0.000	0.125	0.000	1.000	[-0.251, 0.251]	
	Age	0.073	0.130	0.561	0.577	[-0.188, 0.334]	0.35
	General anxiety	0.093	0.137	0.677	0.501	[-0.181, 0.367]	0.26
	MA	-0.293	0.137	-2.140	0.037^*^	[-0.567, -0.019]	1.88
Model 2	Intercept	0.000	0.118	0.000	1.000	[-0.236, 0.236]	
	Age	0.093	0.121	0.767	0.446	[-0.150, 0.336]	0.35
	General anxiety	0.103	0.125	0.825	0.413	[-0.147, 0.353]	0.26
	Math self-efficacy	0.443	0.123	3.607	< 0.001^***^	[0.197, 0.689]	45.32
Model 3	Intercept	0.000	0.126	0.000	1.000	[-0.252, 0.252]	
	Age	0.066	0.131	0.507	0.614	[-0.196, 0.328]	0.35
	General anxiety	0.027	0.130	0.207	0.836	[-0.233, 0.287]	0.26
	Math skills	0.276	0.130	2.128	0.038^*^	[0.016, 0.535]	2.20

Note. *β* = standardized Beta coefficient; SE = Standard Error; *t* = *t*-test; *p* = *p*-value; 95% CI = 95% confidence interval; BF = Bayes Factor

* *p* <.05, *** *p* <.001

The results showed higher levels of MA were negatively associated with Dot-Probe task scores in the congruent condition (*β* = -0.293, *t* = -2.140, *p* =.037, BF_10_ = 1.88), indicating that more anxious subjects tended to respond more slowly to the probe, subsequent to exposure at the same screen location of a math word compared to a neutral one, showing an attentional bias avoidance pattern. However, the Bayes Factor (BF_10_ = 1.88) suggests that the evidence supporting this association is inconclusive (BF_10_ < 3.00). In addition, the results showed that Dot-Probe task scores in the congruent condition were positively predicted by math self-efficacy (*β* = 0.443, *t* = 3.607, *p* <.001, BF_10_ = 45.32) and math skills (*β* = 0.276, *t* = 2.128, *p* =.038, BF_10_ = 2.20). In other words, subjects with lower math self-efficacy and lower math skills tended to respond more slowly to the probe preceded by a math word than a neutral one, showing an attentional bias avoidance pattern. Considering the Bayes Factor, the positive association between Dot-Probe task scores in the congruent condition and math self-efficacy is supported by strong evidence (BF_10_ = 45.32), while the relationship between Dot-Probe task scores in the congruent condition and math skills remains inconclusive (BF_10_ < 3.00).

### Dominance analysis

In order to assess the relative contribution that the predictors have on the Dot-Probe task scores in the congruent condition, we conducted a dominance analysis. Dominance analysis is a statistical procedure for assessing how much each variable relatively contributes to the variance of the dependent variable, evaluating them both independently and in conjunction with all other possible combinations of predictors (Budescu, 1993). This procedure is particularly suitable when examining the relative contribution made by each variable with respect to the dependent variable, especially when predictors are expected to be correlated with each other (Azen & Budescu, 2003). Indeed, the relative contribution of predictors in traditional multiple regression models is typically inferred from the magnitude of their regression coefficients. However, these coefficients can fluctuate depending on which other predictors are included or excluded from the model, making it challenging to accurately determine the relative importance of each variable. In this context, dominance analysis overcomes this limitation by computing the average explained variance of a specific predictor, examining all possible other predictor combinations, providing a more reliable assessment of their relative average contribution to explaining the variance in the outcome (see Azen & Budescu, 2003).

We used the *dominancenalysis* package in R (Bustos & Soares, 2024) to assess the average relative contribution of each predictor. We first specified a full multiple regression model by placing the scores of the Dot-Probe task in the congruent condition as the dependent variable, and age, general anxiety, MA, math self-efficacy and math skills as predictors. Next, we calculated the average contribution of each independent variable in explaining the variance of the dependent variable. This contribution was quantified using the change in the multiple coefficient of determination (Δ*R²*) when the independent variable was added to different submodels, each containing an increasing number of other predictors:

- Level 0: Represents the unique contribution of each predictor when it is included alone in the model, i.e., the variance explained by that single predictor.
- Level 1: Indicates the average additional contribution of the predictor when it is added to a model that already contains another predictor. In other words, it measures how much the explained variance improves compared to considering only the other predictor individually.
- Level 2: Represents the average additional contribution of the predictor when it is added to a model that already contains two other predictors.
- Level 3: Measures the average additional contribution of the predictor when it is added to a model that already contains three other predictors.
- Level 4: Indicates the contribution of the predictor when it is included in the full model, containing all other predictors.

**Fig. 2:**
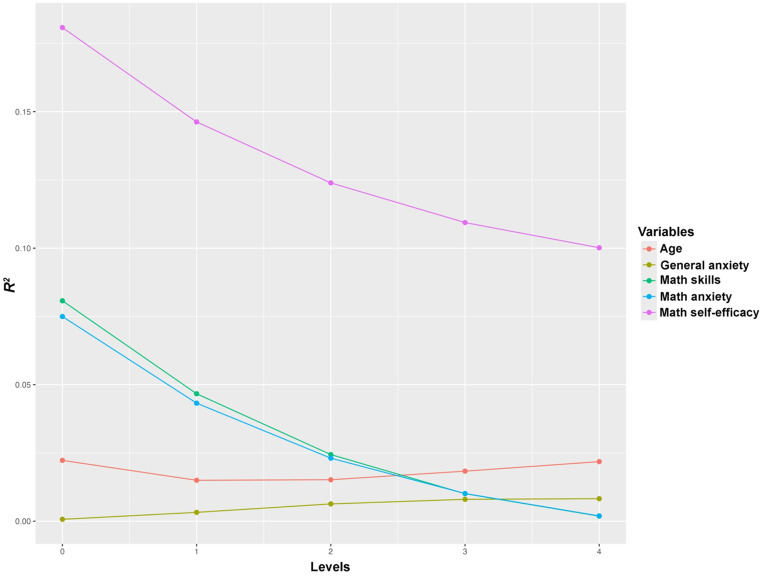
Graphical representation of the dominance analysis conducted on Age, General anxiety, Math skills, Math anxiety and Math self-efficacy. The graph illustrates the average contribution of each predictor to explaining the variance in Dot-Probe task scores in the congruent condition, as measured by *R²* (coefficient of determination). The x-axis levels (0–4) represent the average increase in *R²* when the predictor is added to models that include 0 to 4 additional predictors. Each line shows how the predictor’s contribution varies with the inclusion of other variables in the model

Finally, by averaging the average contributions of each variable across all four levels, we determined the overall relative importance of each predictor in explaining the variance of the dependent variable. In this way, dominance analysis allows us to evaluate not only the individual importance of each predictor but also how its contribution changes in the presence of other variables in the model (see Table 3, and Fig. 2 for a graphical representation).

**Table 3:** Dominance analysis considering as dependent variable dot-probe task scores in the congruent condition. The table displays the average contribution of each predictor to explaining the variance in Dot-Probe task scores in the congruent condition, expressed as *R²* (coefficient of determination). Levels 0 through 4 represent the average increase in *R²* when the predictor is added to models that include 0, 1, 2, 3, or 4 additional predictors, respectively. The “Average” column shows the overall average contribution of each predictor across all possible submodels. Predictors are sorted according to their average unique contribution to dot-probe task scores in the congruent condition

Predictor	*R*-Squared (*R*^2^)					
	Level 0	Level 1	Level 2	Level 3	Level 4	Average
General anxiety	0.001	0.003	0.006	0.008	0.008	0.005
Age	0.012	0.006	0.005	0.005	0.006	0.007
MA	0.075	0.044	0.023	0.009	0.001	0.030
Math skills	0.081	0.049	0.028	0.014	0.008	0.036
Math self-efficacy	0.181	0.144	0.118	0.099	0.086	0.125

Results showed that math self-efficacy explained the largest average variance of Dot-Probe Task scores in the congruent condition (mean *R*^*2*^ = 0.125). On the other hand, results revealed that math skills (mean *R*^*2*^ = 0.036), MA (mean *R*^*2*^ = 0.030), age (mean *R*^*2*^ = 0.007), and general anxiety (mean *R*^*2*^ = 0.005), explained on average only a modest proportion of the variance.

## Discussion

Mathematical knowledge is essential in an increasingly technological and engineered society (Gerardi et al., 2013; Gross et al., 2009; Rivera-Batiz, 1992). However, many people feel uncomfortable when they have to face math related concepts (Layzer Yavin et al., 2022; Organization for Economic Cooperation and Development, 2013). Importantly, these negative attitudes toward math are present already in the first years of primary school (e.g., Caviola et al., 2022; Namkung et al., 2019; Pellizzoni et al., 2022) and seem to manifest also in the way individuals process threatening stimuli. This phenomenon is labelled attentional bias (MacLeod et al., 1986; Mogg et al., 2004). Numerous studies have confirmed attentional bias toward math stimuli in adults (e.g., Pizzie & Kraemer, 2017; Rubinsten et al., 2015) leaving almost unexplored this process in school-age students. For this reason, the aim of the present study was to comprehend the role of emotional-motivational factors (i.e., MA and math self-efficacy) and math skills on attentional bias toward math related words in a sample of primary and middle school students.

In accordance with our starting hypotheses, results showed that higher MA, lower math self-efficacy, and lower math skills were each associated with an attentional bias avoidance pattern toward math-related words, as revealed in the three regression analyses considering the Dot-Probe task congruent condition. These findings are consistent with previous studies conducted among adults, which found an attentional bias toward math-related stimuli and examined the association between MA (Pizzie & Kraemer, 2017; Rubinsten et al., 2015), math skills (Cohen & Rubinsten, 2017) and attentional bias. We also found, in separate regression models, that participants with higher anxiety, lower math self-efficacy, and lower math skills were slower in processing the probe when preceded by a math word compared to when it was preceded by a neutral word, indicating an attentional bias avoidance pattern in the Dot-Probe task congruent condition. Literature has indicated that the attentional bias follows a time course: subjects would first show vigilance and then avoidance towards the threat. Thus, our findings might suggest the involvement of processes that would prompt individuals to avoid threatening math words as a fear reduction strategy (Haft et al., 2019), suggesting the presence of a mechanism similar to those observed in specific phobia (e.g., Salum et al., 2013; Waters et al., 2014). Overall, our results are consistent with evidence in the literature in which avoidance of threatening math stimuli has been shown to occur in math anxious adults (Pizzie & Kraemer, 2017).

However, it is important to point out that our results are in contrast to some studies considering adult samples (e.g., Cohen & Rubinsten, 2017; Rubinsten et al., 2015) where an opposite pattern emerged, i.e., individuals with high MA showed vigilance to threat and exhibited faster RTs when probes appeared in the same location as math stimuli. These inconsistent results can be first explained from a methodological point of view. As suggested by Pizzie and Kraemer (2017), the contrasting results may depend in part on the characteristics of the Dot-Probe task used. Indeed, in studies where robust vigilance to threat was found (e.g., Cohen & Rubinsten, 2017; Rubinsten et al., 2015), the Dot-Probe task required subjects to use significant cognitive resources to process and solve the *prime*, which consisted of, for example, two- or three-digit math operations. On the other hand, in the present study we used a simplified version of the Dot-Probe task with words (math vs. neutral) rather than math operations in order to observe whether children’s attentional bias would be triggered by the math semantic context itself. Additionally, using words allowed us to control for the impact that complex math operations may have on the person’s attitude towards the task. A second possible explanation for these inconsistent results may be the different time duration of exposure to the *prime*. In fact, in accordance with the vigilance-avoidance theory (e.g., Mogg et al., 2004), when primed with a threat stimulus, children might show vigilance at first and then exhibit avoidance of the threatening stimulus. In this sense, the longer the exposure to the threatening stimuli, the more likely the child is to have an influence from processes that could manifest in threat avoidance (Haft et al., 2019). Finally, a developmental viewpoint may also help to explain inconsistent results. According to a meta-analysis by Lisk et al. (2020), considering studies conducted on anxious children, there seems to be a tendency to avoid threatening stimuli over vigilance patterns, especially when considering fear disorders (Salum et al., 2013; Waters et al., 2014). In this context, MA could have the characteristics of a fear disorder, acting on attentional processes similar to specific phobias (Haft et al., 2019; Pizzie & Kraemer, 2017; Salum et al., 2013; Waters et al., 2014).

Dominance analysis findings showed that math self-efficacy made the largest average unique contribution to Dot-Probe task scores in the congruent condition, particularly in relation to attentional bias avoidance patterns. Results may reflect that math self-efficacy plays a primary role in eliciting avoidance patterns observed when individuals are exposed to threatening math stimuli (see Cisler & Koster, 2010; Haft et al., 2019). Previous studies have shown how attention can be modulated by motivational constructs that may also play a role in the orientation of attention (Karademas et al., 2007; Walsh et al., 2018). One possible explanation for why math self-efficacy emerged as the construct with the largest relative contribution in this study could be that the Dot-Probe task captured later-stage attentional bias processes (Mogg et al., 2004). Theoretically, in the short term, attentional bias is driven by more bottom-up, uncontrolled processes, which then give way to more controlled, emotion-regulation attentional processes (Cisler & Koster, 2010; Mogg et al., 2004; Walsh et al., 2018). In this context, math self-efficacy may have influenced emotional regulation processes following exposure to a stimulus perceived as threatening by the student, thereby contributing to the avoidance patterns observed in the Dot-Probe task congruent condition. On the other hand, dominance analysis showed that MA and math skills explain only a modest proportion of the variance of Dot-Probe task scores in the congruent condition. In this context, in line with the vigilance-avoidance theory (e.g., Mogg et al., 2004) anxiety could have affected attentional bias in the early stages after the threatening stimulus is presented, leading the subject to show vigilance patterns (Abado et al., 2020; Bar-Haim et al., 2007; Cisler & Koster, 2010; Mogg et al., 2004). In our task, we exposed the participants to math words for 1000ms, and this duration may have prevented the observation of vigilance patterns that would occur within this timeframe. In other words, it is possible that the Dot-Probe task used in our study specifically detected motivational processes at later stages that helped regulate the child’s behavior in facing the threatening stimuli.

### Limitations

Our research has some limitations. First, we exposed the prime words for a specific time (i.e., 1000ms) in the Dot-Probe task, which may have captured only certain aspects of attentional bias. Future studies will need to look more broadly at the time course of attentional processes to assess how individuals exhibit attentional bias in earlier and later stages. More broadly, future investigations should seek to identify the variability that influences the direction of attentional bias (i.e., vigilance vs. avoidance patterns) by manipulating methodological factors and considering different age groups. Furthermore, considering the incongruent condition of the Dot-Probe task, the score was not related to either MA, or math self-efficacy, or math skills. This represents a limitation of our task, as we had expected to find statistically significant associations with the constructs examined, aligned with an avoidance attentional bias. One possible explanation for this discrepancy with our expectations may lie in the design of our task, which is an adaptation of the traditional Dot-Probe task. In the traditional Dot-Probe paradigm, two prime stimuli are presented in two different positions on the screen, with the probe subsequently appearing in one of those positions. In this setup, the presence of two primes could facilitate spatial attention toward the locations where the probe will later appear. In contrast, in our task, participants were exposed to only one prime stimulus at a time, which, in the incongruent condition, did not predict the location of the probe that would appear on the opposite side of the screen. This may have involved other cognitive processes, such as reorienting attention to the probe on the opposite side of the screen, possibly leading to less consistent Dot-Probe task scores in the incongruent condition. In this context, future studies should exercise greater caution when presenting a single prime stimulus and explore alternative experimental strategies to ensure that children have effectively processed the prime word. Finally, future studies should consider employing longitudinal designs to explore how attentional bias may be reinforced over time by students’ math attitudes and how it may contribute to the persistence of specific forms of anxiety towards the discipline.

#### Constraints on generality

The generalization of the results of the present study must consider several factors that may influence research outcomes. While Dot-Probe task paradigms have been extensively utilized in research and have demonstrated notable correlations with anxiety-related disorders (Abado et al., 2020; Aue & Okon-Singer, 2015; Waters et al., 2014), recent studies and reviews have raised concerns regarding its reliability (Xu et al., 2024), partly due to the heterogeneity of methodological approaches employed in the literature (Chapman et al., 2019; McNally, 2019). Despite ongoing investigations, there remains a lack of consensus on the instrument’s reliability or the optimal methods to improve it. Consequently, it is advisable to interpret and generalize the findings of this study cautiously, with a particular emphasis on its methodological considerations. Firstly, the study was conducted on participants with typical development and medium socio-economic status. For this reason, caution must be exercised in generalizing the results, since emotional manifestations may vary when considering clinical populations or other age groups (Abado et al., 2020; Aue & Okon-Singer, 2015; Lemaire, 2022). Future studies should replicate the present findings on children with high MA, as has been done on adults (e.g., Rubinsten et al., 2015), or on children diagnosed with Specific Learning Disorders (e.g., Haft et al., 2019). Additionally, particular care should be taken when replicating the Dot-Probe task. Our task involved exposing the stimulus for a specific duration (i.e., 1000ms), and different time intervals could potentially alter the results of the present study (Mogg et al., 2004). In regard to the other measures used in the study, we believe that substituting instruments with equally valid and reliable alternatives would be unlikely to change the study’s outcomes. This would be supported by previous research, which has predominantly relied on self-report measures for adults (Cohen & Rubinsten, 2017; Pizzie & Kraemer, 2017; Rubinsten et al., 2015).

## Conclusion

To summarize, our study investigated how attentional bias avoidance patterns toward math stimuli can be influenced by affective, motivational aspects and math skills, advancing literature both from theoretical and practical perspectives. First of all, results showed that besides MA, math self-efficacy and math skills were also associated with an avoidance response toward threatening stimuli in a sample of primary and middle school children. Furthermore, it appears that math self-efficacy has the most robust contribution in predicting attentional bias, suggesting its primary role in explaining threatening stimuli avoidance patterns. The attentional bias would occur rapidly and outside the individual’s awareness and, from a clinical perspective, could be a foundational risk factor for the maintenance of avoidance behaviors toward math. So far, a variety of activities are proposed in the literature to decrease negative emotions evoked by math. However, our results suggest that simple exposure to math stimuli can also evoke attentional bias toward the threatening stimuli. For this reason, mere exposure to math stimuli may not be the optimal choice to reduce negative attitudes towards the discipline. Previous investigation on different math trainings seem to underline exactly this point (Balt et al., 2022; Passolunghi et al., 2020). In fact, making children more aware of math strategies could be more effective in decreasing negative attitudes towards math. Therefore, future works should investigate attentional biases toward math stimuli more deeply, designing interventions aimed at their modulation with the goal of tempering negative attitudes toward math.

## Data Availability

The data supporting this study are openly available on the Open Science Framework (OSF) at https://osf.io/u7ehp/.
